# Elucidation of Short Linear Motif-Based Interactions of the FERM Domains of Ezrin, Radixin, Moesin, and Merlin

**DOI:** 10.1021/acs.biochem.3c00096

**Published:** 2023-05-24

**Authors:** Muhammad Ali, Alisa Khramushin, Vikash K. Yadav, Ora Schueler-Furman, Ylva Ivarsson

**Affiliations:** † Department of Chemistry − BMC, Uppsala University, Husargatan 3, 751 23 Uppsala, Sweden; ‡ Department of Microbiology and Molecular Genetics, Institute for Medical Research Israel-Canada, Faculty of Medicine, The Hebrew University of Jerusalem, Jerusalem 9112102, Israel

## Abstract

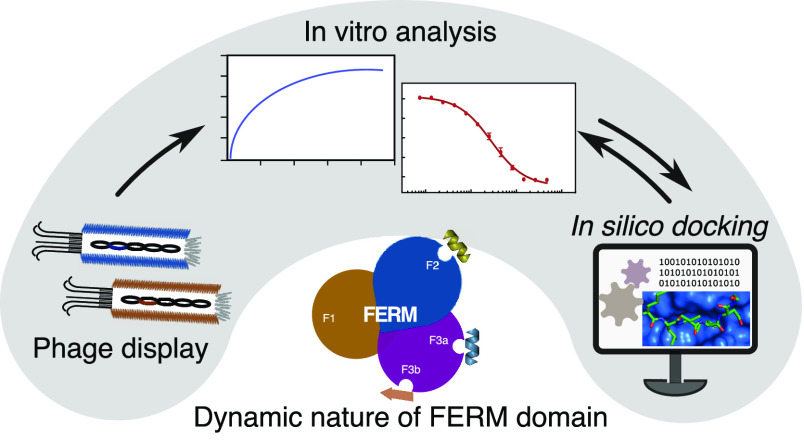

The ERM (ezrin, radixin, and moesin) family of proteins and the related protein merlin participate in scaffolding and signaling events at the cell cortex. The proteins share an N-terminal FERM [band four-point-one (4.1) ERM] domain composed of three subdomains (F1, F2, and F3) with binding sites for short linear peptide motifs. By screening the FERM domains of the ERMs and merlin against a phage library that displays peptides representing the intrinsically disordered regions of the human proteome, we identified a large number of novel ligands. We determined the affinities for the ERM and merlin FERM domains interacting with 18 peptides and validated interactions with full-length proteins through pull-down experiments. The majority of the peptides contained an apparent Yx[FILV] motif; others show alternative motifs. We defined distinct binding sites for two types of similar but distinct binding motifs (YxV and FYDF) using a combination of Rosetta FlexPepDock computational peptide docking protocols and mutational analysis. We provide a detailed molecular understanding of how the two types of peptides with distinct motifs bind to different sites on the moesin FERM phosphotyrosine binding-like subdomain and uncover interdependencies between the different types of ligands. The study expands the motif-based interactomes of the ERMs and merlin and suggests that the FERM domain acts as a switchable interaction hub.

## Introduction

**Figure 1:**
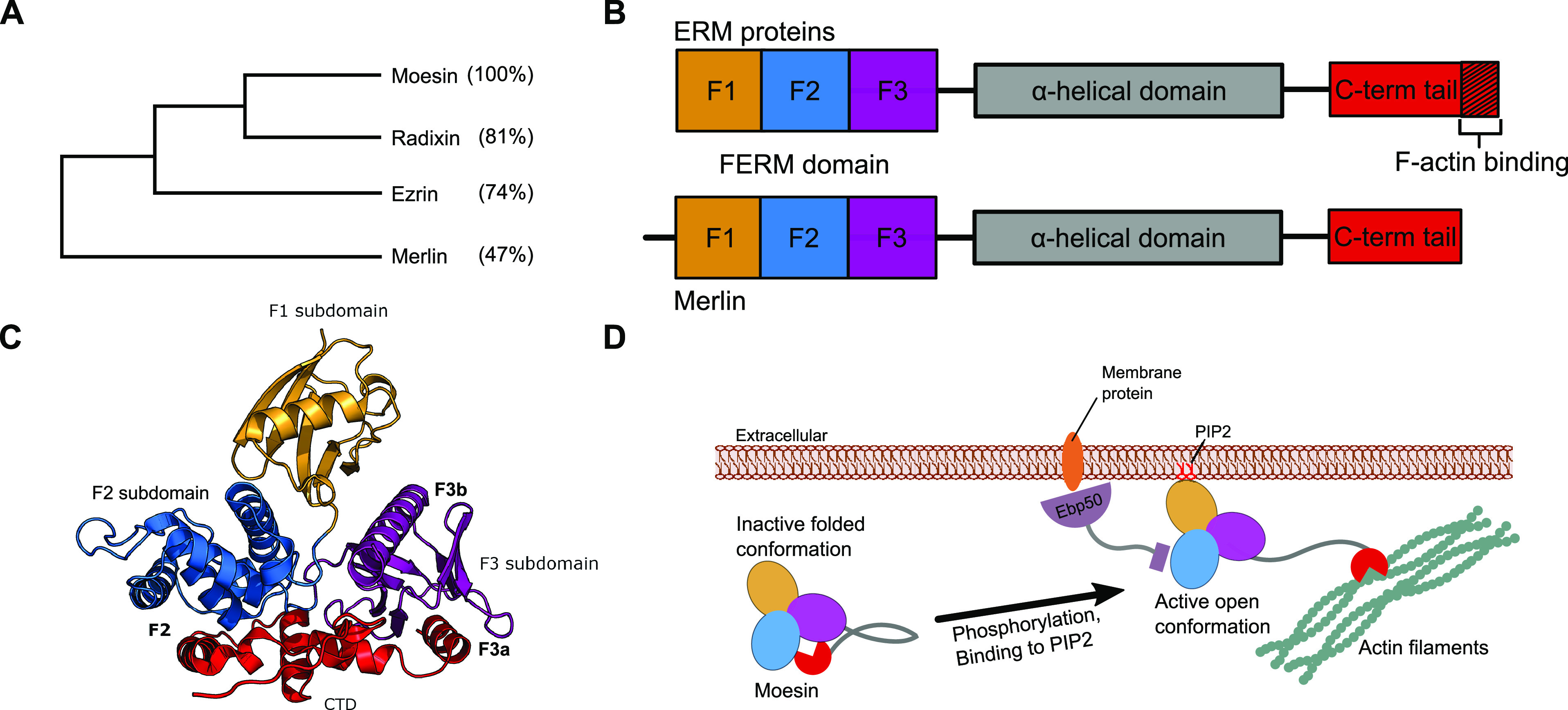
Overview of the ERM and merlin subfamily of the FERM domain containing proteins. (A) Sequence identities between full-length proteins show that merlin is the most distant variant in terms of sequence divergence. (B) Modular architectures of the ERMs and merlin reflect a common N-terminal FERM domain, which is connected through an α-helical domain to the CTD. (C) Structure of moesin in complex with its CTD. The different subdomains and the binding sites are indicated (PDB: 1EF1(5)). (D) In its inactive, closed form, the CTD binds to the FERM domain. Upon phosphorylation and binding to PIP2, the two domains are free to interact with their partners: The FERM domain interacts with SLiM-containing target proteins, mainly in the plasma membrane’s vicinity, while the CTD domain binds actin filaments. Panel (B, D) inspired by Fehon, McClatchey, and Bretscher.^1^

Ezrin, radixin, and moesin (collectively referred to as the ERMs) are membrane-associated proteins that provide linkage between membrane and actin cytoskeleton.^1,2^ The ERMs are closely related (Figure 1A) and share an N-terminal FERM domain (F for 4.1 protein, E for ezrin, R for radixin, and M for moesin), followed by a region with α-helical propensity and a C-terminal domain (CTD) that binds to F-actin (Figure 1B). The protein merlin, encoded by the NF2 gene, is closely related to the ERMs (Figure 1A). Merlin is a well-known tumor suppressor protein and an upstream regulator of the Hippo pathway.^3^ Compared to the ERMs, merlin lacks the C-terminal F-actin binding region (Figure 1B) and has distinct tissue localization and function.^4^ Here, we focus on the proteins that bind with short linear motifs (SLiMs) to the FERM domains of ERMs and merlin.

The FERM domain has a cloverleaf-like structure with three subdomains, F1, F2, and F3 (Figure 1C), forming a ubiquitin-like, an acyl-CoA binding protein-like, and a phosphotyrosine binding (PTB)-like domain, respectively.^5^ The FERM domain serves as a hub that binds to SLiM-containing proteins and membrane phospholipids.^2,6,7^ Several distinct SLiM-binding sites have been found distributed over the different subdomains, including two closely located but distinct binding pockets on the F3 domain: F3a and F3b (Figure 1C). The FERM domain ligands can be classified based on their binding pocket preferences (Table 1). Following this classification, the -LxEI**-** containing peptides (where x = any amino acid) from the serine/threonine-protein kinases LATS1/2 belongs to class F2 as they bind to subdomain F2,^8^ and the [L/I]Fxx[L/F]-coo- motif found in the C-termini of the Na(+)/H(+) exchange regulatory cofactor EBP50 (NHERF1) and NHERF2 belongs to class F3a.^9,10^

**Table 1:** Overview of ERM and Merlin Peptide Ligands with Known FERM Binding Sites Based on Crystal Structures of Complexes

FERM domain protein	ligand	peptide sequence^a^	binding pocket	PDB code(s)
**radixin**	MMP14	RR**LLY**CQ	F1	3X23(11)
	EBP50 (NHERF1)	**MDW**SKKNE**LF**SN**L**	F3a	2D10(10)
	NHERF2	**MDW**NRKRE**IF**SN**F**	F3a	2D11(10)
	CD43	TGA**L**T**L**S	F3b	2EMS(12)
	CD44	KKK**L**V**I**N	F3b	2ZPY(13)
	ICAM2	**R**TGT**Y**G**V**LA**A**	F3b	1J19(14)
	PSGL-1	KTHM**Y**P**V**RNY	F3b	2EMT(15)
	NEP	**M**D**IT**D**IN**	F3b	2YVC(16)
**moesin**	CTD		F2, F3a	1EF1(5)
	EBP50 (NHERF1)	MDWSKKNEL**F**SN**L**	F3a	1SGH(9)
	CD44	KKK**L**V**I**N	F3b	6TXS
	CRB	ATR**G**T**Y**S**P**SA	F3b	4YL8(17)
**merlin**	CTD		F2, F3a	3U8Z,^18^7EDR,^19^4ZRJ^8^
	LATS1	KA**L**QE**I**RNS**L**LP**F**	F2	4ZRK(8)
	LATS2	KA**L**RE**I**RYS**L**LP**F**	F2	4ZRI(8)
	DCAF1	D**I**I**L**S**L**N^b^	F3b	3WA0,^20^4P7I^21^

a Bold letters indicate residues implicated in binding.

b A longer β-hairpin motif was suggested by Li and coauthors.^21^

The functions of the ERM proteins are regulated by autoinhibitory interactions between their C-terminal region and their FERM domain. In the closed conformation, the C-terminal region of the proteins blocks both F2 and F3a sites.^6,7,9^ Phosphorylation and binding to PIP2 activates the protein (Figure 1D).^22−25^ Allosteric regulation has further been suggested between the different binding sites. In particular, binding of a peptide from EBP50 to the F3a site of radixin caused a conformational change that narrowed the F3b site.^10^

Here, we explore the peptide binding of the FERM domains of the ERMs and merlin by screening them against a proteomic peptide-phage display library (ProP-PD) that tiles the intrinsically disordered regions of the human proteome,^26^ define consensus motifs that bind to distinct pockets, and investigate the interplay between distinct types of ligands using a competitive peptide binding assay and computational modeling.

## Materials and Methods

### Plasmids, Cloning, and Mutagenesis

Synthetic genes encoding for human merlin FERM (P35240; amino acids 22–312) and radixin FERM (P35241; 5–295) domains were commercially synthesized (ThermoFisher) and cloned into pETM33 using NcoI and EcoRI restriction sites. pGEX4T Moesin (P26038) FERM was a gift from Vijaya Ramesh (Addgene plasmid # 1163^27^). pGEX4T1 Ezrin (P15311) FERM domain was a kind gift from Volker Gerke.^28^ Human NOP53-HA-pcDNA was a kind gift from Ronit Sarid.^29^ The NOP53 gene was polymerase chain reaction (PCR)-amplified and cloned into CMV10 using HindIII and EcoRI. RRBP1-GFP was kindly provided by Alexander F. Palazzo.^30^ HA-HIF1-alpha-pcDNA3 was a gift from William Kaelin (Addgene plasmid # 18949^31^). pcDNA3 LATS1 was a gift from Erich Nigg (Addgene plasmid # 41156^32^). LATS1 was PCR-amplified and cloned into CMV10 using NotI and BamHI restriction sites. GFP-FAM83G-pcDNA was a kind gift from Gopal Sapkota.^33^ HA-PAK6-pcDNA was kindly provided by Michael Lu.^34^ ZNF622 was a gift from Hyunjung Ha.^35^ All ligands for the pull-down experiment were mutated at the binding site following the QuickChange mutagenesis protocol. The moesin FERM domain was mutated in two combinations of M285A/H288A and K211A/I238A. Mutations were made sequentially by following the QuickChange mutagenesis protocol. Sequences of all obtained, cloned, and mutated constructs were confirmed using Sanger sequencing. Gene names and associated Uniprot accession numbers related to the constructs used are provided in Table 2.

**Table 2:** Gene Names and Associated Uniprot Accession Numbers for Constructs Used

gene name	Uniprot accession
BOC	Q9BWV1
BTBD7	Q9P203
CAMK2B	Q13554
EBP50	O14745
EZR	P15311
FAM83G	A6ND36
GCM2	O75603
HIF1A	Q16665
ICAM3	P32942
KIRREL3	Q8IZU9
LATS1	O95835
MISP3	Q96FF7
MSN	P26038
NF2	P35240
NOP53	Q9NZM5
PAK6	Q9NQU5
PDCD6IP	Q8WUM4
RDX	P35241
RIMS1	Q86UR5
RORA	P35398
RRBP1	Q9P2E9
TBX4	P57082
ZNF622	Q969S3

### Protein Expression and Purification

Expression constructs encoding the FERM domains of merlin, moesin, ezrin, and radixin were transformed into *E. coli* BL21(DE3). For expression, 2xYT medium (1.6% tryptone, 1% yeast extract, and 0.5% NaCl) was inoculated with a fresh overnight culture of the transformed cells and grown until the OD_600_ reached 0.8. Protein production was induced using 0.3 mM isopropyl β-d-1-thiogalactopyranoside (IPTG) by incubating cells overnight at 18 °C while shaking. Next day, cells were harvested by centrifuging at 7000 × *g* for 10 min. For purification, the pellet was homogenized using PBS (137 mM NaCl, 2.7 mM KCl, 10 mM Na_2_HPO_4_, 1.8 mM KH_2_PO_4_, pH 7.4) containing lysozyme, DNase I, cOmplete protease inhibitor cocktail (Roche), 1% Triton X-100, and 2 mM β-mercaptoethanol. Cells were lysed for 30 min while shaking at 4 °C followed by sonication for 5 cycles of 30 s. Cell debris was removed by centrifuging at 20,000 × *g* for 45 min, and the supernatant containing His-GST-FERM domains was incubated with glutathione (GSH) sepharose resin (Cytiva) for 1 h. Beads were collected and washed on a column. The protein was eluted using 10 mM reduced GSH in PBS, pH 8.0 and dialyzed to PBS overnight using dialysis SnakeSkin tubing for subsequent use in phage selections and pull-down experiments. Protein size and purity were analyzed by SDS-PAGE electrophoresis. The quality and stability of wild-type (WT) and mutant proteins were evaluated as described below.

For fluorescence polarization (FP) experiments of merlin FERM, the GST tag was removed using HRV3C protease by incubating the enzyme (100 units/10 mg protein) with protein overnight at 4 °C in the dialysis buffer (20 mM HEPES, pH 7.4, 150 mM NaCl, 0.05% Tween-20, and 3 mM DTT). The following day, the cleaved GST and the protease were removed using reverse Ni-IMAC, and the protein was concentrated to the working concentration. For FP experiments using moesin FERM, thrombin (Sigma) was used to cleave the GST tag from moesin FERM domain on GSH sepharose beads (Cytiva) and incubated overnight at 4 °C under gentle rotation. Next day, the beads were collected by gentle centrifugation, while the supernatant containing thrombin and moesin FERM domain was passed through a HiTrap benzamidine column (Cytiva). The protein was then eluted using a high salt (0.7–1 M NaCl) concentration. The buffer was then exchanged using the PD-10 desalting column (Cytiva) into FP buffer (20 mM HEPES, pH 7.4, 150 mM NaCl, 0.05% Tween-20, and 3 mM DTT).

### Protein Quality and Stability

Purified moesin FERM domain was subjected to gel filtration using Superdex 200 (Cytiva) columns, and the monomeric species were collected. During experiments, proteins were further subjected to various quality checks. W130i dynamic light scattering (DLS) (Avid Nano) was used before each set of moesin FP experiments to check if the protein was monomeric. Analytical gel filtration (Superdex 300GL (Cytiva)) also confirmed the monomeric state of moesin. The stability of WT and mutant moesin was analyzed by thermal unfolding using 5 μM of each protein on Tycho NT.6 (nanoTemper). Inflection temperatures (*T*_i_) were identified, and the relative stability was analyzed by comparing with WT moesin in the optimized buffer (20 mM HEPES, pH 7.4, 150 mM NaCl, 0.05% Tween-20, and 3 mM DTT), later used for FP measurements.

### ProP-PD Selections

Phage selections were performed for 4 days using a phage library that displays 16 amino acid long peptides tiling the intrinsically disordered regions of the human proteome on the p8 protein of the M13 phage.^26^ Three or more independent selections were performed for each bait protein. For each replicate selection, 25 μg of target protein (GST-tagged FERM domains of merlin, moesin, ezrin, and radixin) and GST control were immobilized overnight to a 96-well maxisorp plate well while shaking at 4 °C. Next day, the wells were blocked using 0.5% bovine serum albumin (BSA) in PBS for 1 h. The naïve phage library containing 10^11^ colony forming units was precipitated using 1/5th volume of PEG/NaCl (20% PEG8000 and 0.4 M NaCl) followed by centrifugation at 10,000 × *g* for 10 min and dissolved in 100 μL of PBS for each well. Control wells were washed four times using 200 μL of PBS containing 0.05% Tween-20 (PBST) and incubated with phages for 1 h at 4 °C with shaking to remove the nonspecific binders. The target wells were washed as before, and the phage solution was transferred to them and incubated for 2 h at 4 °C. Unbound phages were removed by 4× washing with 200 μL of PBST. The bound phages were eluted by adding 100 μL of log phase *E. coli* OmniMax for 30 min at 37 °C. M13KO7 helper phages (10^11^ pfu/mL) were added to each well and incubated again at 37 °C for 45 min. Each hyper-infected bacterial culture was transferred to 1 mL of 2xYT containing kanamycin (50 μg/mL), carbenicillin (100 μg/mL), and 0.3 mM IPTG and incubated overnight with shaking at 37 °C. Following day, bacteria were pelleted by centrifugation at 5000 × *g* for 10 min, and the supernatant was used for phage precipitation as before. Phages were consequently dissolved in 1 mL of PBS.

To determine the progress of the phage selections, a sandwich ELISA was performed. Briefly, 10 μg of target and control proteins were immobilized overnight in a 96-well maxisorp plate. Wells were blocked using 0.5% BSA. 100 μL of phage solution from each binding-enriched phage pool was added to the control and target wells and incubated for 1 h at 4 °C with shaking. The wells were washed four times using PBST and were incubated with anti-M13 coat HRP-conjugated antibody (1:5000) dilution for 1 h. The unbound antibody was washed away as before, and TMB substrate was added and allowed to develop the blue color. Reaction was stopped using 0.6 M H_2_SO_4_, and the absorbance intensity was determined at 450 nm.

Peptide-coding regions of binding-enriched phage pools were amplified and barcoded using PCR. The DNA pools were analyzed using NGS on the Illumina platform (MiSeq), and the results were demultiplexed and analyzed using a pipeline described elsewhere.^36^ The DNA sequences were translated to amino acid sequences, resulting in peptides associated with sequencing read counts. Confidence levels were based on four metrics (occurrence of peptides in replicate selections, identification of regions with overlapping peptides, high NGS counts, and the presence of consensus motif). Peptides that meet two to four of these criteria were considered of medium/high confidence, as benchmarked by Benz et al.^36^

### Fluorescence Polarization Assay

Synthetic peptides were obtained at 95% purity (GeneCust) and were dissolved in FP buffer (described earlier). To obtain direct binding saturation data, a dilution series of moesin FERM domain was prepared using the FP buffer, and then an equal volume of 10 nM FITC-labeled peptide was added to each sample. After mixing, the FP signals were recorded using SpectraMax iD5 (Molecular Devices). Data were analyzed with GraphPad Prism version 7.0.0 for MacOS (GraphPad Software, San Diego, California USA). A quadratic equation for equilibrium binding^37^ was used to fit the obtained data.
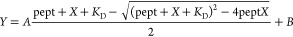
where *pept* indicates the probe peptide concentration (5 nM), *X* indicates the protein concentration, the constant *A* being the signal amplitude divided by probe peptide concentration, and *B* is the plateau value. Direct binding experiments were performed and analyzed the same way in the presence of saturating concentration of unlabeled peptides. In these experiments, the unlabeled peptide was used at a concentration approximately 100 times of the *K*_D_ values (80–150 μM concentrations).

For the FP competition experiments, a precomplex containing moesin FERM at 2 × *K*_D_ concentration (for respective probe peptide) and 10 nM of FITC-labeled probe peptide was made in the FP buffer. 25 μL of this premix was added to 25 μL of titrated target peptide. FP signals were determined as described earlier. Data were fitted to a sigmoidal dose–response (variable response; GraphPad Prism) model, and the Hill coefficient was found to be around 1 for all experiments. All measurements were performed at least in triplicates.

### Cell Culture and Pull-Down Assays

HEK293 cells (Sigma:85120602) were cultured using Dulbecco’s modified Eagle medium (Gibco) supplemented with 10% fetal bovine serum and NEAA (Gibco) in a humid environment at 37 °C while maintaining 5% CO_2_. For pull-down experiments, the cells were transiently transfected with tagged-target proteins using Fugene HD (Promega) following manufacturer’s recommendations. Cells were allowed to grow and express the proteins for 48 h post transfection. Cells were washed with ice-cold washing buffer (PBS, Halt Protease Inhibitor Cocktail, pH 7.4) and then incubated with lysis buffer (50 mM Tris–HCl, pH 7.4, 150 mM NaCl, 10 mM sodium pyrophosphate, 10 mM sodium orthovanadate, 10 mM sodium fluoride, 1× cOmplete EDTA-free protease inhibitor tablet, and 0.5% Nonidet P-40) for 30 min at 4 °C with gentle shaking. Cell debris was removed by centrifuging at 16,000 × *g* for 20 min at 4 °C, and protein concentration was determined using the BCA protein assay (Pierce). Supernatant containing 0.5 mg of total protein was mixed with the target FERM domain or GST (negative control) and one of the beads: GSH magnetic agarose beads (Pierce), magnetic GFP-Trap (Chromotek), and anti-FLAG M2 magnetic beads (Sigma) according to manufacturer’s recommendations. This mixture was incubated overnight with end-over-end rotation at 4 °C. Following day, the beads were collected and washed with lysis buffer three times. Samples were eluted using SDS-sample buffer by boiling at 95 °C for 5 min.

The eluted samples were resolved by SDS-PAGE using 4–20% gradient gels and then transferred to the nitrocellulose membrane using the Trans-Blot Turbo transfer system (Biorad). Following the blocking for 1 h in blocking buffer (5% milk in TBST), immunoblotting was performed using 1:2000 anti-FLAG (sigma), 1:2500 anti-GFP (ab6556), 1:2000 anti-HA (Sigma), 1:2500 anti-GST (Sigma), and 1:2000 anti-Myc (ab9106) antibodies followed by 1:5000 anti-rabbit HRP-conjugated secondary antibodies (GE) in blocking buffer. Membranes were exposed to Amersham ECL western blotting detection reagent (Cytiva) for a minute, and signals were imaged using the ChemiDoc Imaging system (Bio-Rad). Proteins with mutated putative binding sites were also immuno-precipitated and blotted similarly.

### Structure Preparation for Docking Simulations

For our simulations, we used the available solved structures of moesin FERM domains, including (i) the free moesin FERM domain (PDB ID 6TXQ), (ii) the structure of moesin FERM domain bound to its own CTD, which occupies the F3a and F2 sites (PDB ID: 1EF1(5)), (iii) moesin with the crumbs CTD bound at the F3b site and in the cleft between the F3 and F1 lobes (PDB ID: 4YL8(17)), and (iv) moesin with the CD44-derived peptide bound to the F3b site (PDB ID: 6TXS).

For peptide–protein interaction modeling, two moesin crystal structures solved with different peptides were used: (1) F3b-bound (bound to CD44, PDB ID 6TXS) and (2) F3a-bound (bound to CTD at the F3a site, PDB ID 1EF1). For the latter, we removed the crystal contact peptide occupying the F3b site, which may bias the simulation toward the supposedly closed F3b site, and relaxed the structure using the Rosetta FastRelax protocol^38^ restraining heavy atoms to their native coordinates. Using this restrained “relax” protocol, we attempted to remove any bias generated by the crystal contact.

### Global Blind Peptide Docking Using PIPER-FlexPepDock

Global docking was performed using the PIPER-FlexPepDock protocol.^39^ In brief, the peptide conformation is represented as an ensemble of fragments extracted from the PDB, based on sequence and (predicted) secondary structure using the Rosetta Fragment picker (with the vall2011 fragment library).^40^ These fragments are mutated to the target peptide sequence with the Rosetta fixed backbone design protocol.^41^ 50 fragments are rigid-body docked onto the receptor protein using the PIPER rigid body docking program. The top 250 models for each fragment are then further refined using the Rosetta FlexPepDock protocol,^42^ including receptor backbone minimization, and top-scoring models are clustered. In this study, all Rosetta simulations were performed using Rosetta version 2019.14. The protocol is freely available for noncommercial use as an online server: https://piperfpd.furmanlab.cs.huji.ac.il.

### Peptide Threading with Rosetta FlexPepBind

The FlexPepBind protocol^39^ uses a template structure of a protein-peptide interaction to thread a list of peptides onto the template and refine each using FlexPepDock. The top-scoring among all models is selected as prediction. In this study, structural minimization only was used to refine the complexes, including both peptide and receptor backbone minimization. In some cases, where the threaded sequence was longer than the template peptide, the peptide sequences were threaded onto possible overlapping windows in the template.

### Modeling Conformational Changes with the Rosetta FastRelax Protocol

The Rosetta Relax protocol is used for full-atom refinement of protein structures. In this study, the FastRelax protocol was applied with default parameters (no constraints were enforced) to open a pocket by superimposing the ligand to its binding site on an unbound structure. In the simulation, 200 decoys were generated, and the top scoring model was taken for further analysis.

## Results and Discussion

### Identification and Characterization of FERM Domain Ligands

**Figure 2:**
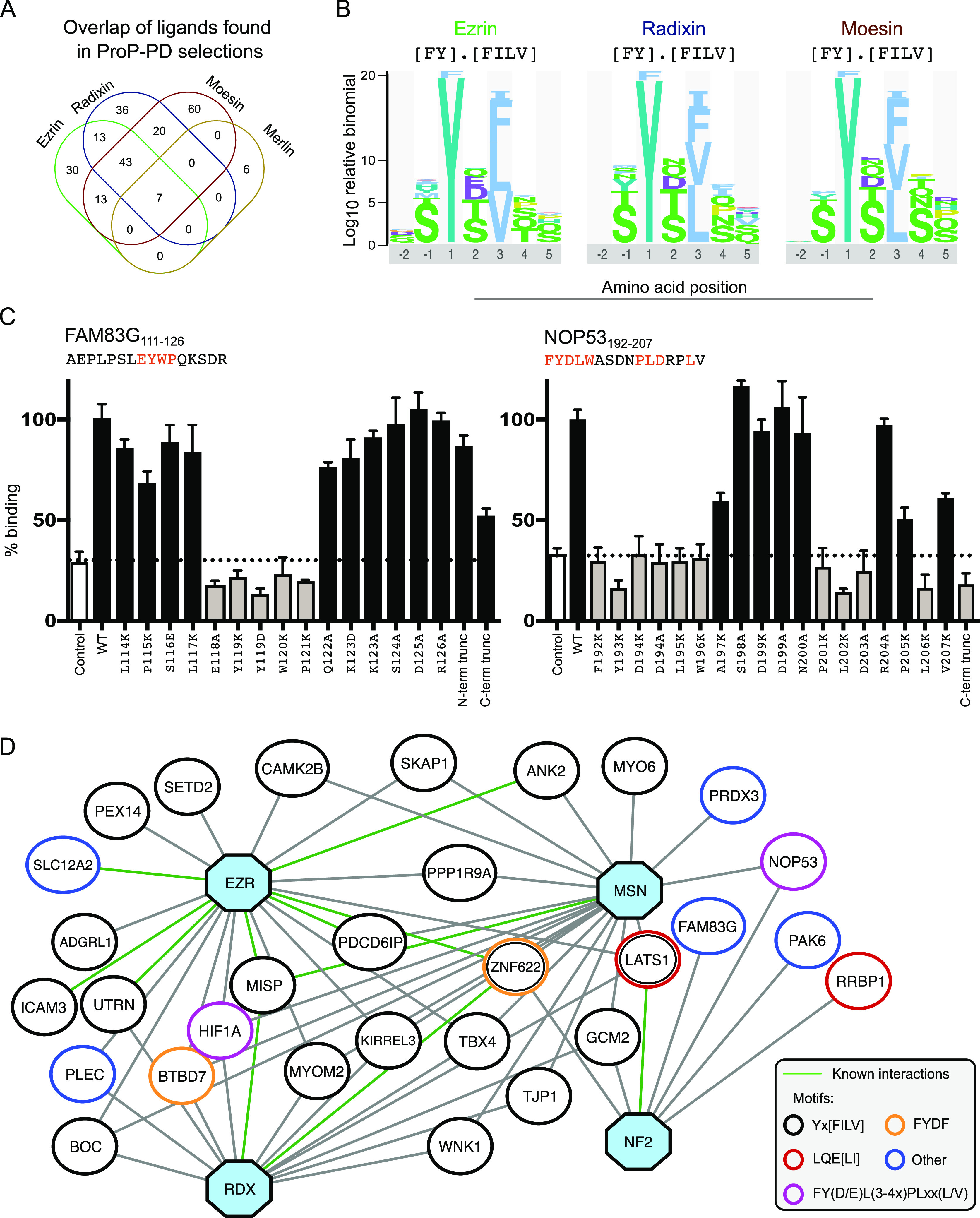
Overview of ProP-PD results, motifs, and ligands identified for the FERM domains of the ERMs and merlin. (A) Overlap of high/medium confidence peptide ligands identified through phage selections against the ERM proteins and merlin (Tables S1–S4). (B) Consensus binding motifs of the ERM ligands generated by PepTools.^24^ (C) Key amino acids in merlin binding FAM83G and NOP53 peptides identified through mutational analysis. The effects of the mutations on binding were evaluated by clonal phage ELISA against immobilized GST-tagged merlin FERM domain. The binding was assessed by the ratio of the *A*_450_ values detected for the immobilized target protein (GST-tagged merlin) to that of the background (GST). The results were normalized to 100% binding of the respective WT peptide. As an extra negative control (indicated “control)”, a clonal phage ELISA was performed for the same proteins using an M13 phage displaying no peptide. (D) Network of a selected set of protein–protein interactions. Shown interactors include proteins that share biological functions with the baits that are unlikely to occur by chance based on GO term analysis (Tables S1–S4). The baits ezrin (EZN), radixin (RDX), moesin (MSN), and merlin (NF2) are indicated as blue octagon. Interactions supported by results from other studies are indicated with green edges. The binding motifs of the interactors are indicated by the node color.

We used the purified FERM domains of the ERMs and merlin as baits in four rounds of selections against a previously described ProP-PD library that displays peptides representing intrinsically disordered regions of the human proteome.^26^ Binding-enriched phage-pools were analyzed by NGS. The sequences were translated into peptides and were assigned with confidence scores following an established protocol (0: no confidence; 1: low, 2–3: medium and 4: high) based on four metrics, namely, the (i) occurrence of peptides in replicate selections, (ii) identification of an amino acid region with overlapping peptides, (iii) high NGS counts, and (iv) presence of consensus motifs.^36^ We focused on the high/medium confidence set of ligands (Tables S1–S4). Between 106 and 143 unique peptides were found for each of the ERM FERM domains, with a substantial overlap of ligands between the domains (Figure 2A). For the merlin FERM domain, the analysis identified only eight medium confidence peptides (Table S4). To gain more information on merlin ligands, we tested a set of lower confidence peptides for binding using clonal phage ELISA (Figure S1), which confirmed the interactions of additional five ligands. Consensus motifs were generated based on the binding-enriched peptides using the SliMFinder algorithm,^43^ which showed that the peptide set obtained for the ERM FERM domains is dominated by ligands with an apparent Yx[FILV] motif (Figure 2B).

For merlin, there were not enough peptides to establish a consensus motif. We therefore characterized the binding determinants of two ligands, FAM83G_111–126_ (the most enriched ligand in the phage display against merlin FERM) and NOP53_192–207_ (previously shown to interact with merlin^44^), using a mutational analysis evaluated by clonal phage ELISA. A combination of lysine and alanine scanning was used for a clear readout in the relatively insensitive binding assay. For FAM83G_111–126_, a central EYWP stretch was necessary and sufficient for binding (Figure 2C) as mutations of any of these residues abrogated binding, and truncation of the N-terminal region (AEPLP deletion) or the C-terminal region (KSDR deletion) only had minor effects on binding. For NOP53_192–207_, the interaction required an extended FYDLWxxxxPLDxxL stretch. This was confirmed by the finding that a C-terminal truncation of the peptide (deletion of PLDRPLV) conferred loss of binding.

On the protein level, we performed a gene ontology (GO) term enrichment analysis of the ERM domain ligands which revealed an enrichment of proteins associated with cell–cell adhesion, nervous system development, and synapse organization, as well as proteins involved in DNA binding (Table S5). The protein–protein interaction set contained 18 peptides from previously reported interactors (Figure 2D). Among the known interactors, we note interactions between ezrin and ankyrin-2 (ANK2) previously found through proximity labeling mass spectrometry (AP-MS).^34^ We found that ezrin FERM binds three different ANK2 peptides (_2545-_EVS**Y**E**V**TPKTTDVSTP-_2560_, _2950-_HTTSFHSSEV**Y**S**V**TIT-_2965_, and _3761-_PEESSLEYQQE**Y**F**V**TT-_3776_). We further found a peptide from the mitotic interactor and the substrate of PLK1 (MISP_595–610_), a protein which has previously been found to bind to the ERMs through high-throughput affinity purification coupled to MS^45^ and yeast-two-hybrid.^46^ The set of merlin ligands contained two peptides from known interactors, LATS1^8,47^ (_73-_THHKA**L**Q**EI**RNSLLPF-_88_) and NOP53 (_192-_**FYDL**WASDN**PLD**RP**L**V_-207_).^44^ Moreover, we found a merlin-binding RRBP1 peptide (_1365-_RAATRLQELLKTTQEQ_-1380_). RRBP1 has previously been found to be proximal to merlin through proximity biotinylation.^25^ Notably, the merlin-binding RRBP1 peptide contains a _1370_-LQEL-_1373_ stretch that is similar to the F2 binding motif in LATS (_78-_LQEI_-81_). Identification of peptides found in previously reported interactions support the quality of ligands identified through the ProP-PD selections and provide detailed binding site information for the known protein–protein interactions.

### Validation of Motif-Based Interactions through Affinity Measurements and Pulldowns

**Figure 3:**
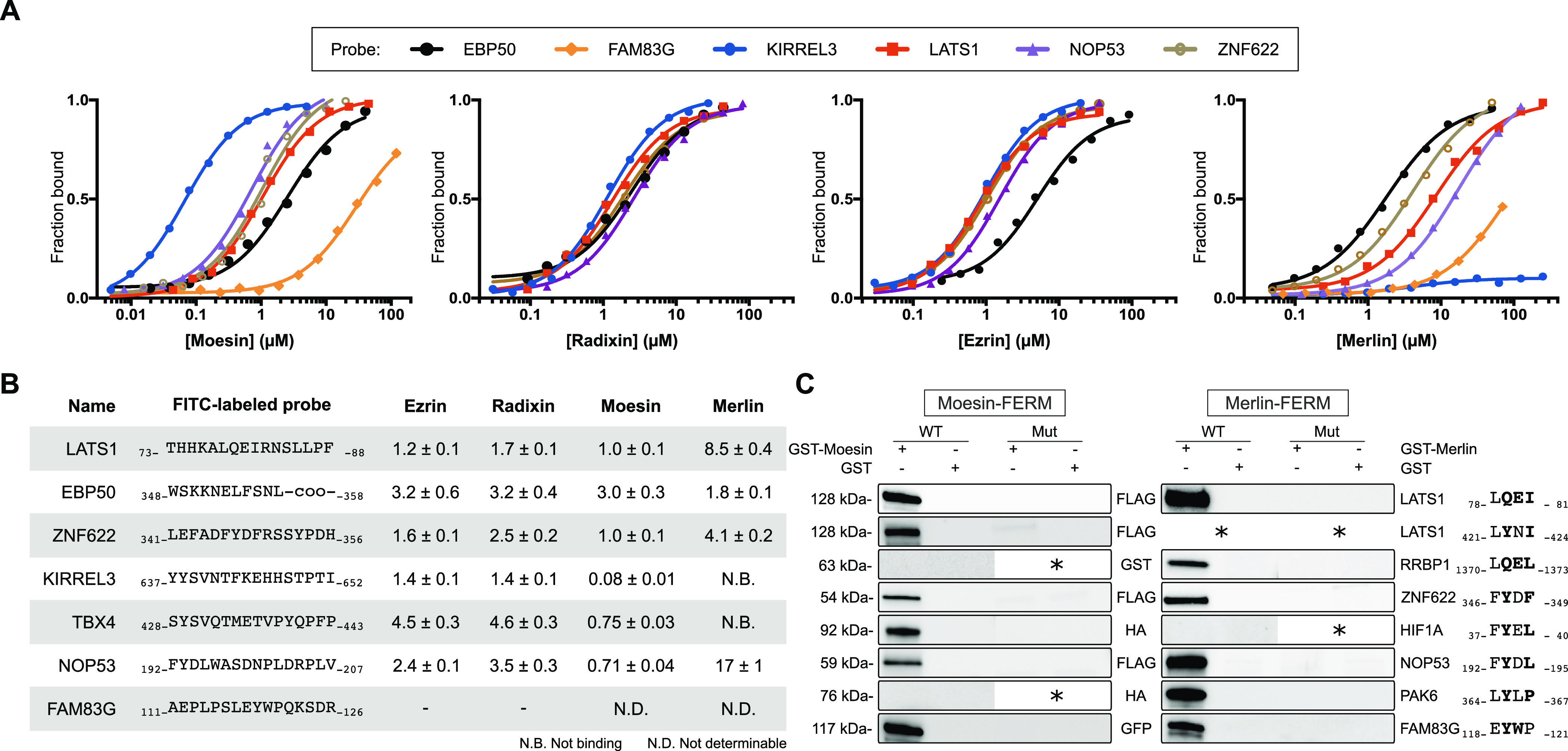
Determination of affinities through direct binding FP experiments and validation of interactions with full-length proteins through GST-pulldowns. (A) FITC-labeled probe peptides (5 nM) were titrated with increasing concentrations of moesin, ezrin, radixin, and merlin FERM domains. The results were fitted to a quadratic equation for 1:1 binding (number of technical repeats (*n*) = 3). The raw FP data are available in Figure S2. (B) Summary of *K*_D_ values as determined in (A). Most of the tested ligands bind to ERM FERM domains with low-micromolar *K*_D_ values and to merlin with lower affinity. (C) GST-pulldowns of WT and motif-mutant full-length proteins. GST-tagged moesin or merlin FERM domains were used to pull down WT or mutant proteins transiently expressed in HEK293 cells. Mutated amino acids are indicated to the right (bold residues were mutated to alanine). Note that LATS1 has two moesin binding motifs. Results shown are representative of at least two replicated experiments. * indicates not tested.

We determined the affinities (*K*_D_) of the FERM domains for a selected set of ligands, namely, KIRREL3_637–652_ (KIRREL3: Kin of IRRE-like protein 3), TBX4_428–443_ (TBX4: T-box transcription factor TBX4), ZNF622_341–356_ (ZNF622: Zinc finger protein 622), NOP53_192–207_, FAM83G_111–126_, LATS1_73–88_, and EBP50_348–358_ (Figure 3A) by a direct binding FP assay using fluorescein (FITC)-labeled probe peptides. The three first peptides were selected as variants of the Yx[FILV] motif (Figure 2B). The two peptides from NOP53_192–207_ and FAM83G_111–126_ have distinct motifs as shown by the clonal phage analysis against merlin (see above, Figure 2C), and LATS1_73–88_ and EBP50_348–358_ were included as they are known ligands binding to the F2 and F3a pockets, respectively (structures for complexes with these ligands have been solved, see Table 1). The *K*_D_ values measured for interactions with the ERMs were in the low micromolar range, except for the nanomolar affinity moesin-KIRREL3_637–652_ interaction. For merlin, EBP50_348–358_ was the highest affinity ligand, followed by ZNF622_341–356_, LATS1_73–88_, and NOP53_192–207_. Notably, the Yx[FILV] containing ligands KIRREL3_637–652_ and TBX4_428–443_ did not bind merlin within the concentration range tested (Figure 3B). This is in line with previous studies reporting that the merlin and the ERMs have partially overlapping but distinct specificities.^17,21^ We further determined the affinity between merlin and RRBP1_1365–1380_ (*K*_D_ 9.7 ± 0.8 μM; Figure S2) and found it to be similar to merlin’s affinity for the F2 binding LATS_73–88_ peptide (Figure 3B).

An additional set of interactions between the ERMs and three Yx[FILV] containing ligands (BOC_1000–1015_, CAMK2B_13–28_, and GCM2_274–289_) were affinity determined through a competitive FP assay where the probe peptide FITC-KIRREL3_637–652_ was outcompeted by increasing concentrations of unlabeled peptides (Figure S3). These ligands were found to bind the ERMs with lower affinities (Table 3). Moreover, we determined the affinities of moesin FERM domain for an additional set of six Yx[FILV] containing ligands, of which the YxV containing MISP_595–610_ peptide bound with the highest affinity. Finally, we determined the affinity of HIF1A_37–52_ for moesin FERM domain using FITC-NOP53_192–207_ as the probe as the peptides share high sequence similarities (FY(D/E)L(4-5x)PLxxx(V/L)) and found the interaction to be of high affinity (Table 3).

**Table 3:** Affinities of the FERM Domains of the ERMs for a Representative Set of Ligands Determined through Competition FP Experiments Using FITC-KIRREL3 as the Probe Peptide (except for HIF1A)^a^

	unlabeled peptides in competition with FITC-KIRREL3	ezrin *K*_D_′ (μM)	radixin *K*_D_′ (μM)	moesin *K*_D_′ (μM)
**BOC_1000–1015_**	L**Y**T**L**PDDSTHQLLQPH	8.0 ± 0.1	17 ± 2	18 ± 1
**CAMK2B_13–28_**	E**Y**Q**L**YEDIGKGAFSVV	26 ± 0.6	6.8 ± 0.4	96 ± 4
**GCM2_274–289_**	S**Y**E**L**ANPGYTNSSPYP	205 ± 11	85 ± 3	38 ± 3
**ICAM3_516–531_**	SGS**Y**H**V**REESTYLPLT	-	-	113 ± 3
**LATS1_421–436_**	L**Y**N**I**SVPGLQTNWPQS	-	-	149 ± 5
**MISP_595–610_**	ITGS**Y**S**V**SESPFFSPI	-	-	2.3 ± 0.1
**PDCD6IP_853–868_**	S**Y**P**F**PQPPQQSYYPQQ	-	-	35.2 ± 0.2
**RIMS1_1405–1420_**	M**Y**T**L**EHNDGSQSDTAV	-	-	564 ± 5
**RORA_185–200_**	T**Y**N**I**SANGLTELHDDL	-	-	134 ± 3
**HIF1A_37–52_**	**FYEL**AHQL**PL**PHN**V**SS			3.0 ± 0.4^b^

a - Not measured.

b Determined using FITC-ZNF622 as the probe peptide.

To further validate the found interactions, we performed GST-pulldown using moesin and merlin FERM domains as baits (Figure 3C). These experiments confirmed that moesin and/or merlin FERM domains interact with full-length FAM83G, ZNF622, NOP53, HIF1A, PAK6, LATS1, and RRBP1 in a SLiM-dependent way, as validated using motif-mutants of the proteins in parallel pulldowns. The proteins have overlapping but distinct specificities for the FERM domains, such that an interaction with HIF1A was only confirmed for moesin, and PAK6 and RRBP1 interactions were only confirmed for merlin, which was consistent with the ProP-PD selection results (Table S4). In the case of LATS1, we validated the importance of the previously known FERM binding region LATS_78–81_. In addition, we also found a hitherto unknown moesin binding motif (_421-_LYNI_-424_) that binds to moesin with lower affinity (Table 3) but is necessary for efficient pulldown of LATS1 by the moesin FERM domain (Figure 3C).

### YxV Motif Binds to the F3b Binding Site as Evidenced by Docking and Mutagenesis

**Figure 4:**
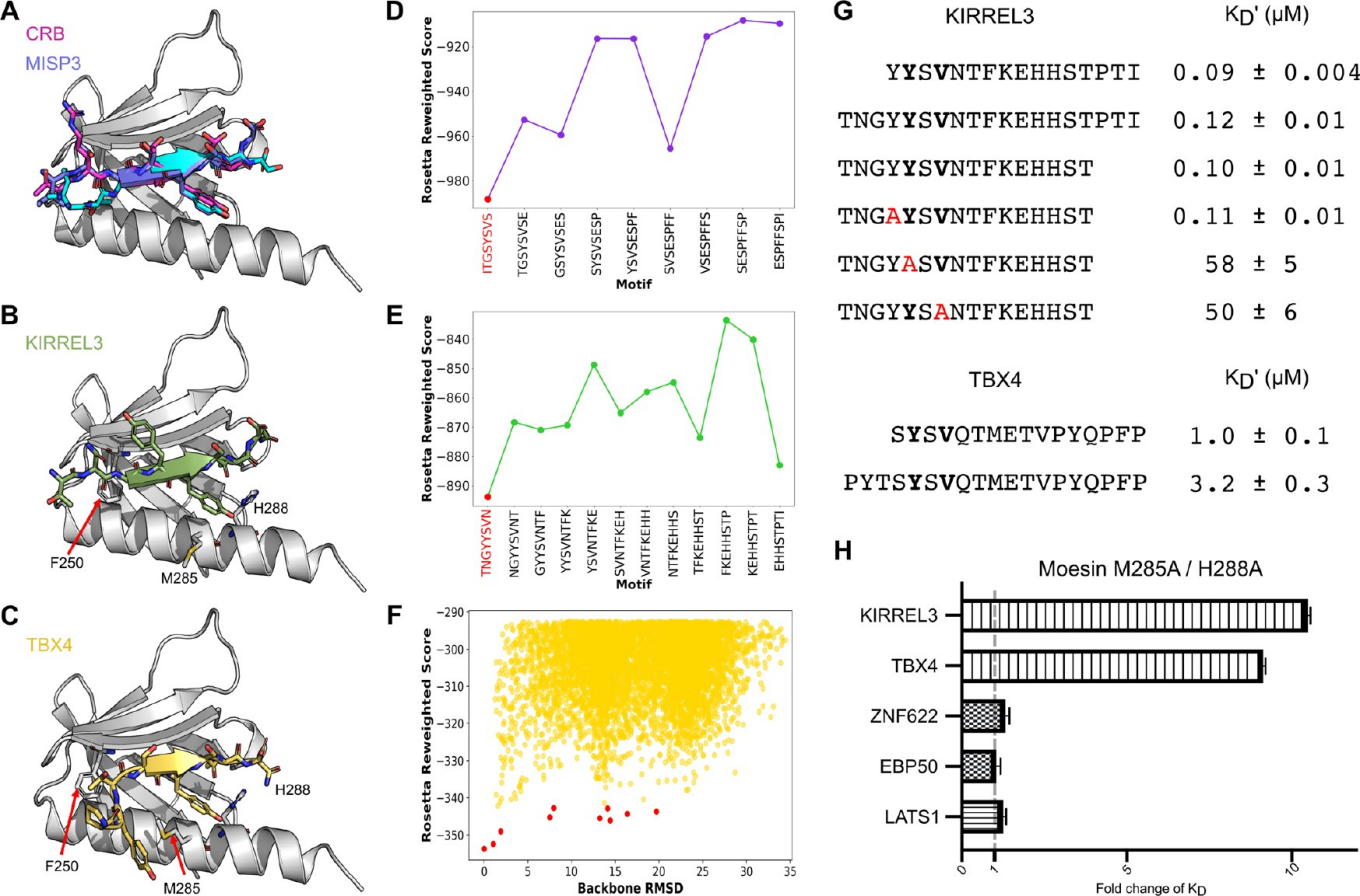
MISP3, KIRREL3, and TBX4 top-scoring peptide models bind the F3b pocket adopting similar conformations as the crumbs (CRB) peptide in the F3b binding pocket. (A) Bound crumbs peptide (magenta, PDB 4YL8), MISP3 top-scoring models from FlexPepBind threading and PIPER-FlexPepDock global docking are shown in purple and cyan, respectively. (B and C) Top-scoring KIRREL3 peptide model from threading and TBX4 peptide model from global docking are shown in green and yellow, respectively. The supposedly crucial residues that interact with the peptides are labeled. (D and E) Threading binding energy landscapes of MISP and KIRREL, respectively. The identified motif is highlighted in red. (F) Global docking simulation binding energy landscape of TBX4, in which the lowest energy models converge toward the F3b site. Top 10 cluster representatives of the simulation are shown with red dots. The RMSD is calculated relative to the lowest scoring structure. (G) Affinities determined through competitive FP-based affinity measurements using variants of the KIRREL3 peptide (top) or the TBX4 peptide (bottom) for competition using FITC-KIRREL3_637–652_ as probe. The mutational analysis validated the importance of the key YxV residues (mutated position in red), while N-terminal extensions of the peptides had no or minor effects on binding. (H) Fold change in the binding affinity of moesin FERM M285/H288A as compared to the WT (*K*_D_^M285/H288A^/*K*_D_^wt^) for six different peptides, as determined by direct binding of FITC-labeled peptides (see Table S7 for complete data).

We next used moesin as a model protein to explore how the peptides bind to the FERM domains. We applied the Rosetta FlexPepDock peptide modeling suite^42^ in two ways: (i) To independently locate the binding motif within the 16 amino acid long peptides found through the phage display, we applied the FlexPepBind protocol^48^ in which different, overlapping peptide sequences are threaded onto a solved protein-peptide complex to identify the best binder. (ii) To confirm the binding site on the FERM domain, we applied the blind, global docking PIPER-FlexPepDock protocol^39^ using the identified motifs. We first analyzed the binding of the MISP_595–610_, KIRREL3_637–652_, and TBX4_428–443_ peptides (Figure 4) that share a YxV motif with the peptides from ICAM2 and crumbs, which have been cocrystallized bound to the F3b site of radixin (PDB 1J19(14)) and moesin (4YL8^17^), respectively. Starting with MISP_595–610_, we applied the FlexPepBind approach using two existing F3b bound structures (PDB codes 4YL8 and 6TXS(17)) as templates and threaded the sequence onto the peptides solved in these structures. The results converged for both simulations, identifying the ITGS**Y**S**V**S sequence as the best ligand for the given binding site (Figure 4A,D). To reaffirm this binding mode, we globally docked the peptide motif onto the FERM domain using PIPER-FlexPepDock. The top-scoring structure identified in this simulation hit the same site as the one identified by threading in a very similar binding conformation (Figure 4A). MISP_595–610_ has a glycine at the p-2 position of the motif (-**G**S**Y**S**V**-), mimicking the glycine at the p-2 position of the ICAM2 and the crumbs peptides which allows tight packing of these peptides with moesin residue F250 (Figure 4B,C).^17^

Threading and global docking of the KIRREL3_637–652_ and TBX4_428–443_ peptides failed to yield conclusive results. We therefore extended the peptides at the N-terminus to include three upstream residues (KIRREL3 TN**G**Y**Y**S**V**N and TBX4 PYTS**Y**S**V**Q). For KIRREL3, the simulations converged in the same way as for MISP: the TNGY**Y**S**V**N sequence identified by the FlexPepBind simulation was ranked best in the global simulation, adopting the same binding conformation as in threading (Figure 4B,E). Mutational analysis of the peptide evaluated by affinity determinations confirmed the importance of the tyrosine at position p1 and valine at position p3 for binding (Figure 4G). As the docking suggested that the presence of a glycine at the p-2 position would improve the affinity, we further tested if extending the KIRREL3 peptide N-terminally would increase the affinity but found it to have no effect. For the TBX4 peptide, threading failed to identify any specific motif. However, global docking of the PYTS**Y**S**V**Q peptide placed it in a very similar position as the previous two peptides, with the N-terminus bulging out to accommodate the larger threonine residue (Figure 4C,F). In the case of TBX4, we found that extending the peptide N-terminally with the sequence PYT reduced the affinity (Figure 4G).

To validate the models on the receptor side, we generated a mutant moesin FERM domain (M285A and H288A) and determined the effect on the affinity. The mutants were designed based on computational alanine scanning^49^ that identified hotspot residues at the binding interface that would significantly affect the binding of the peptides, with a minor effect on the stability of the protein (Table S6A). Consistent with the predictions, the mutations conferred significant loss of affinity of the moesin FERM domain for the YxV containing peptides of the TBX4 and KIRREL3 (Figure 4H). Importantly, the mutations had no or minor effects on binding of the other types of peptides tested (LATS1, EBP50, ZNF622, and NOP53), supporting that of the tested peptides the YxV containing ligands bind specifically to the F3b pocket (Figure 4H and Table S7). The computational and experimental analyses thus show that only the YxV containing ligands bind to the F3b pocket.

### FYDF Containing Peptides Bind to the F3a Pocket

**Figure 5:**
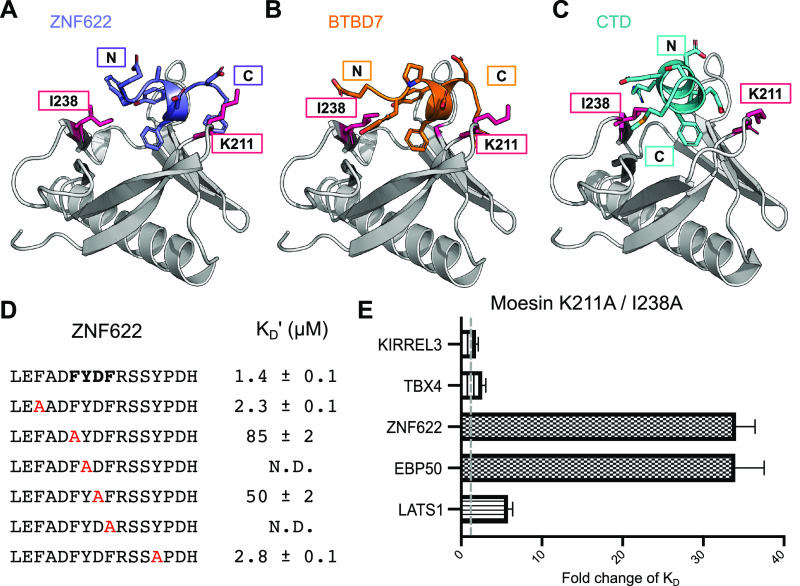
Structural modeling suggests that ZNF622 and BTBD7 bind the moesin FERM domain at F3a using the same binding pocket as moesin CTD and EBP50. Models of the interactions of (A) ZNF622 and (B) BTBD7 suggest a perpendicular conformation, both using aromatic side-chains to fill the same hydrophobic pocket. As expected, this distinct conformation is only identified by a global docking simulation but not by threading. (C) Fragment of moesin CTD bound at the same site (PDB code: 1EF1). (D) Affinities determined through competitive FP-based measurements using WT FITC-labeled ZNF622 as probe. The results highlight the importance of core FYDF motif residues in peptide binding. (E) Fold change in affinity of the moesin K211A/I238A as compared to the WT-moesin for binding with five different peptides. Affinities were determined by direct binding using respective FITC-labeled probes.

We next focused on the two peptides ZNF622_341–356_ and BTBD7_940–950_ that share a FYDF stretch. Global docking of ZNF622_341–356_ onto the full moesin FERM domain positions the 10 top scoring models at the F3a site or in the cleft between the F3 and F1 domains (Figure S4). Docking the peptides onto the isolated F3 domain located the peptide in the F3a and F3b binding sites. However, the previously described mutational analysis of the F3b site showed that binding of ZNF622_341–356_ was unaffected by the mutations introduced in the F3b site (Figure 4G), and the combined results thus suggest that the peptides bind to the F3a site. The F3a bound peptide models did not converge to one single conformation but rather adopted two distinct orientations: one similar to the part of the moesin CTD bound at F3a and another perpendicular to it (Figure 5A). Notably, the hydrophobic side-chains of the top-scoring models of the bound ZNF622_341–356_ occupied the same sites as the CTD of moesin despite overall different peptide conformation (Figure 5C). Mutational analysis of the peptide confirmed that the FYDF stretch is crucial for binding (Figure 5D).

Global docking of BTBD7_940–950_ on the full moesin template structure predicted the cleft between F3 and F1 binding lobes as the most probable binding site. In contrast, docking of the peptide onto the isolated F3 subdomain of the moesin placed most top-scoring models onto the F3a site, although no single defined binding conformation stood out. Although the BTBD7 (EYP**DFYDF)** and ZNF622 (EFA**DFYDF**) peptides share high similarity, PSIPRED^41^ predicted the ZNF622 but not the BTBD7 peptide to form a helix, which can be explained by the presence of a proline upstream of the motif. Consequently, most of the starting fragments used for BTBD7 docking were in extended conformation. Assuming that both adopt a similar local conformation, we repeated our simulation with defined helical fragments. This simulation gave very similar results for BTBD7 to those obtained for the ZNF622 peptide (Figure 5B).

To validate the model, we identified two mutations using computational alanine scanning that would perturb binding (K211A and I238A, Table S6B). A double mutant moesin FERM K211A/I238A was constructed, and the affinity of this mutant for ZNF622 was found to be more than 30-fold lower than the affinity of the WT (Figure 5E), supporting that the peptide binds to the F3a site. The affinity for the known F3a binding EBP50 was similarly affected by the mutations. In contrast, the affinity for the F3b binding KIRREL3 and TBX4 peptides were unchanged, and only minor effects were observed for the F2 binding LATS1 peptide (Table S7). The results thus support that the FYDF containing peptides bind to the F3a site.

### Unveiling the Interplay between Different Ligands

**Figure 6:**
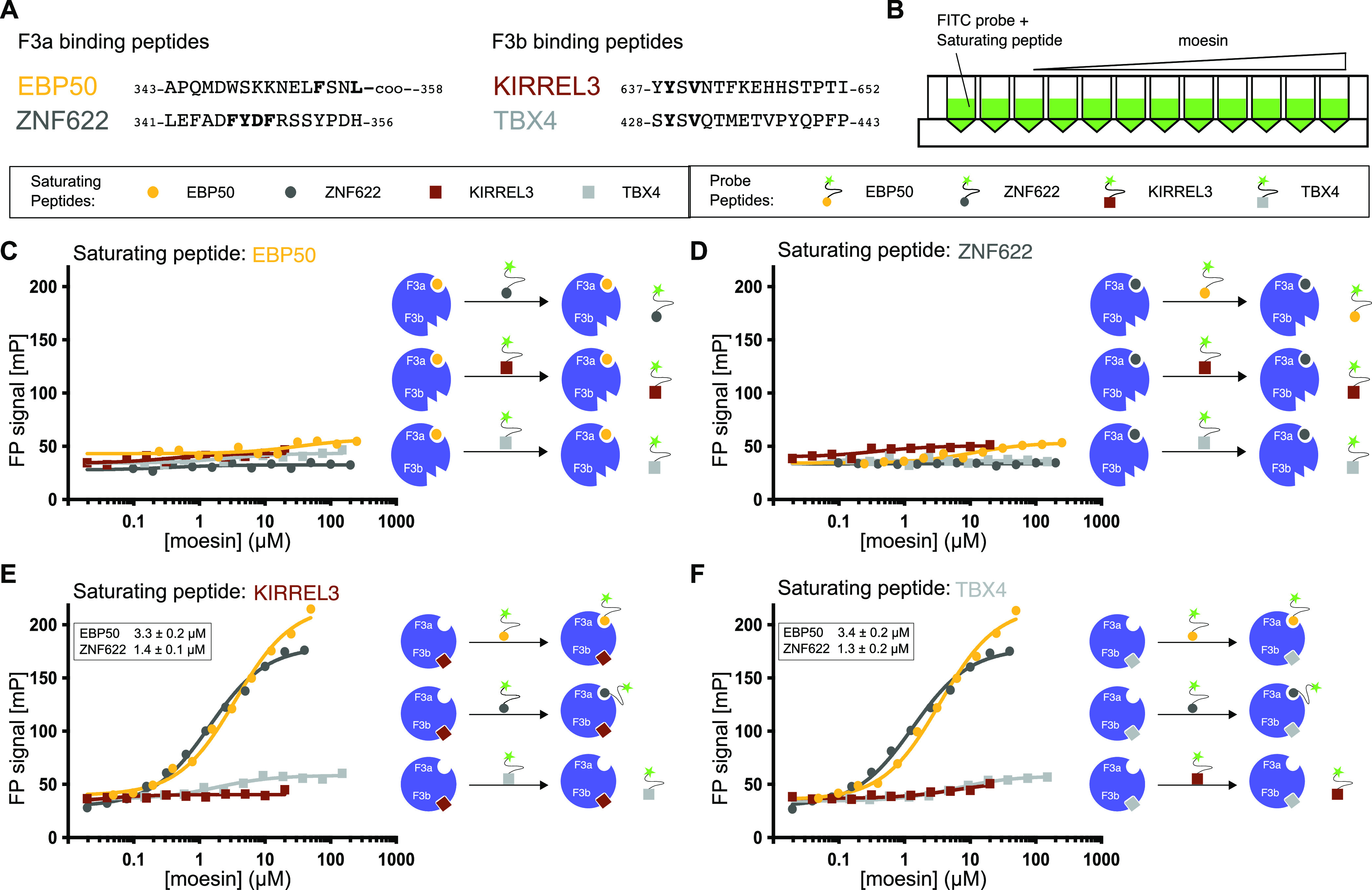
Interplay among different ligands binding to moesin FERM domain F3a and F3b pockets. (A) Representative peptides from the ligand set that bind to F3a and F3b binding pockets show the presence of consensus motifs in the peptide sequence. (B) Schematic of the competition experiment design to evaluate the interplay between moesin FERM binding peptides. Binding of a FITC-labeled probe peptide was monitored in the presence of high saturating concentrations of unlabeled competing peptides, as shown in (C–F). (C and D) When the F3a binding peptides of EBP50 (C) or ZNF633 (D) are in excess, they block binding of all FITC-labeled probe peptides. (E and F) When the F3b binding peptides of KIRREL3 (E) or TBX4 (F) are in excess, they block binding of FITC-labeled probe peptides to the F3b site, but FITC-EBP50 and FITC-ZNZ622 can still bind to the F3a site. Schematics next to saturation plot (C–F) illustrate hypothetical explanations for the observed competition for each combination of titration of labeled peptide with moesin FERM domain in the presence of a saturating concentration of unlabeled peptide binding either to pocket F3a (EBP50 and ZNF622) or F3b (KIRREL3 and TBX4).

We next explored the interplay between the F3a and F3b ligands by determining the affinity of FITC-labeled model peptides in the presence of high concentrations of the competing ligands (Figure 6A,B). We found, as expected, that high concentrations (80 μM each) of the unlabeled F3b ligands (KIRREL3_634–652_ and TBX4_428–443_) blocked binding FITC-KIRREL3_634–652_ or FITC-TBX4_428–443_ to the F3b site but not binding of FITC-EBP50_348–358_ or FITC-ZNF622_341–356_ to the F3a site (Figure 6E,F). In contrast, we found that the excess of the unlabeled F3a ligands EBP50_343–358_ or ZNF622_341–356_ blocked binding to both the F3a and F3b sites (Figure 6C,D). The asymmetric competition could potentially be explained by an allosteric model where binding to the F3a site induces a conformational change of the protein that reduces the affinity for F3b ligands. The model is similar to the previously suggested allosteric inhibition of the F3b site conferred by EBP50 binding to the F3a site of radixin,^10^ although the asymmetric competition is difficult to explain.

### FERM F3 Subdomain Dynamics Affects the Affinity of the Ligands

**Figure 7:**
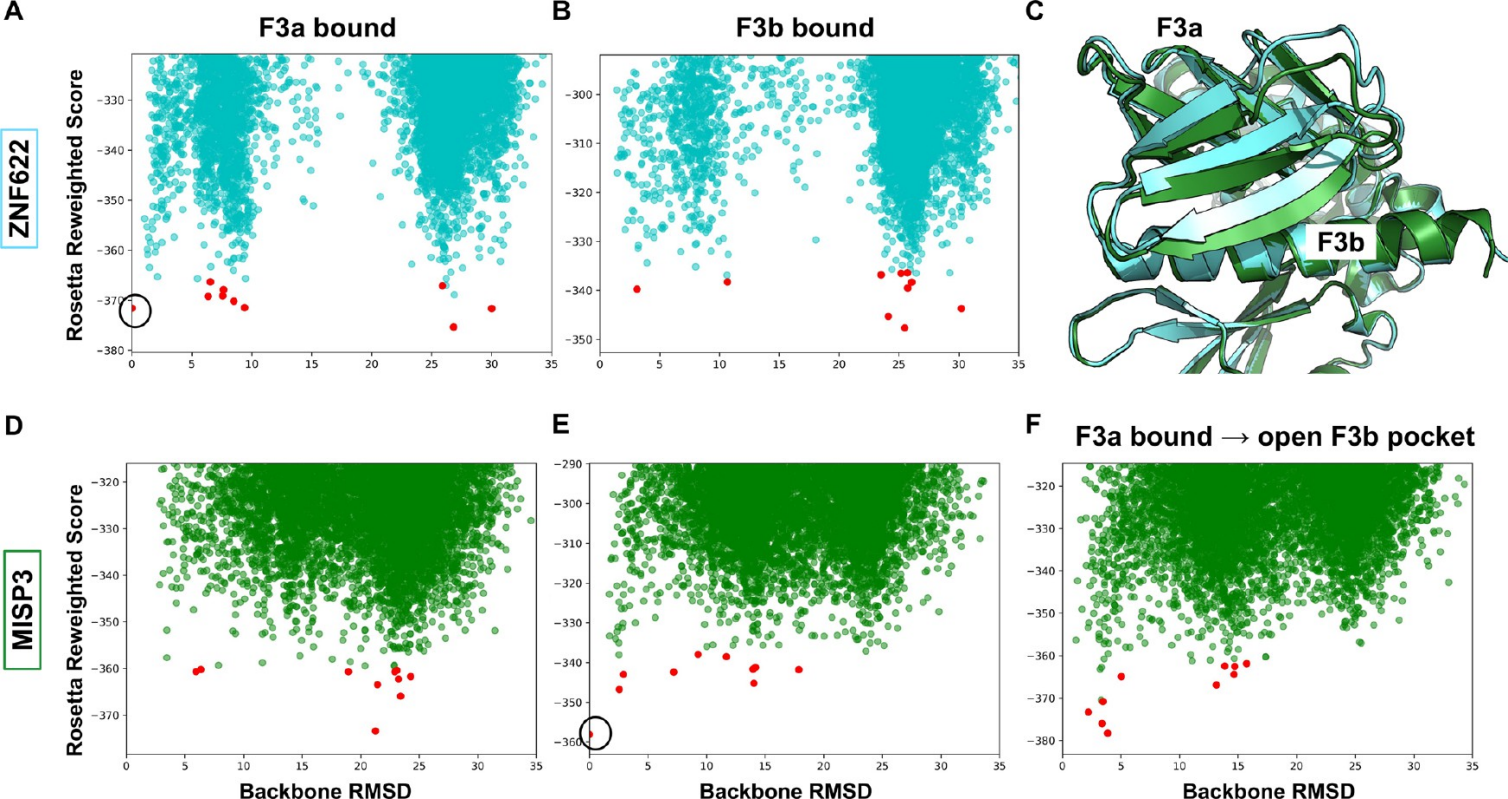
Energy landscapes of simulations using different receptor structures. (A, B) Energy landscape sampled by the ZNF622 peptide: The F3a site is accessible both in F3a bound (A) and F3b bound (B) structures. (C) Comparison of the F3a bound (green) and F3b bound (cyan) structures shows conformational change depending on the occupied binding site. (D, E) Energy landscapes demonstrate that the F3b site is inaccessible in the F3a bound FERM structure: Docking of the MISP peptide onto the F3b-bound (D), F3a-bound (E), and F3a-bound relaxed structure with the superimposed peptide at the F3b binding site (Figure S6) (F) highlights the importance of conformational change for the identification of the F3b binding site. Cluster centers are shown with red dots. Only the F3 subdomain was used in the simulations. The highlighted circles in (A) and (E) show reference models used for RMSD calculations (structures shown in Figures 5A and 4A, respectively).

To evaluate the potential allosteric communication between the F3a and F3b sites, we analyzed how the use of structures cocrystallized with ligands in distinct binding sites affect peptide docking. We started with the ZNF622 peptide and tested whether it would still reach the F3a binding site if starting from a receptor structure cocrystallized with an F3b ligand (6TXS), removing the bound peptide for the simulation. The simulation identified F3a-bound ZNF622 structures among the top ten models (Figure 7B) that are very similar to those from simulation starting from a corresponding F3a-bound receptor structure (1EF1) (Figure 7A). Conversely, for the MISP3 peptide predicted to bind at the F3b site (Figure 4A), we checked whether the peptide would still reach its binding site when a F3a bound structure was used for the simulation (1EF1). This simulation was not able to localize MISP3 to the F3b site (the best peptide backbone RMSD among the top ten cluster-representative structures is 5.9 Å away from the predicted binding conformation, Figure 7D) despite successful identification of the F3b site when a bound receptor conformation was used as the starting template (Figure 7E). We were able to mimic the opening of F3b by relaxing an F3a bound structure in the presence of a superimposed F3b bound peptide (Figure S5). Using that structure, a bound conformation was observed among the top-scoring structures at the F3b site (backbone RMSD of 2.2 Å from the top-scoring result of the bound simulation; Figure 7F). This emphasizes the importance of opening the F3b binding site prior to docking.

To explore the generality of the influence of binding to the F3a site on the binding ability of F3b, we performed a cross-docking analysis with ligands known to bind to the two pockets (i.e., with available solved structures: CD44 as a F3b ligand and the CTD tail as a F3a ligand) using F3b-bound and F3a-bound structures. For CD44, the energy landscape shows no more top-scoring models within the F3b site in the F3a bound structure (see Figure S6) due to F3b pocket closure (see Figure 7C). In turn, no effect on the ability to model CTD binding to the F3a site was observed when using the F3b bound structure (Figure S6). These data demonstrate how the F3a binding peptides can outcompete all other binders including those binding at a distinct pocket (i.e., F3b) by inducing a conformational change at the binding site, while binding to F3b will not affect binding to F3a.

## Concluding Remarks

In this study, we explored the SLiM-based interactomes of the FERM domains of the ERMs and merlin. We identified a large number of ligands binding to the ERM FERM domains and a limited set of merlin ligands. The results support that the ERMs and merlin FERM domains recognize partially overlapping and partially distinct ligand sets. Among the distinct ligands, we note, for example, that moesin but not merlin binds to a SLiM found in HIF1A and that merlin but not moesin binds RRBP1 (Figure 2). The identified ligands provide a rich resource for exploring the molecular function of the ERM proteins in cell–cell adhesion as well as in transcriptional regulation. The latter might provide clues to the nuclear functions of the ERMs and merlin.^50,51^

The datasets contain distinct classes of FERM domain ligands (Figure 2D). We find that the ERMs are capable of binding a large number of Yx[FILV] containing ligands. The Yx[FILV] motif is similar to the previously described ligands found bound to the F3b site (Table 1), and the YxV containing peptides from MISP, KIRREL3, and TBX4 were consistently found to bind to that site through modeling and mutational analysis. We further found a limited set of FYDF-containing ERM ligands, which share some specificity determinants with the Fxx[FL] containing F3a binding peptides from EBP50 and NHERF2. In line with this, we confirmed through modeling and experimental validations that the FYDF-containing peptides of ZNF622 and BTBD7 bind to the F3a pocket. In addition to the discussed motifs, the ProP-PD derived dataset contain peptides with alternative motifs, such as the F2 binding motif of LATS1, the extended FYDLWxxxPLDxxL motif found in NOP53, and the EYWP motif found in FAM83G. Much research remains to be done until we fully understand the enigmatic interactions of the versatile FERM domains.

We further found that there is a complex interplay between binding to distinct sites on the F3 domain of the moesin FERM domain, similar to the previously described allosteric regulation of radixin FERM domain.^10^ Using the information obtained from the existing structures, we built high-resolution models using Rosetta FlexPepDock-based methods and generated refined models of the interactions. These provide a structural basis for two distinct ways of interplay between binding partners: (i) direct competition for the same binding site and (ii) allosteric intradomain communication between the F3a and F3b sites. Using the unconstrained Rosetta FastRelax protocol, we were further able to model the conformational changes inside the domain caused by ligand binding to the F3a site. The known closed conformations of the ERMs and merlin involve the intramolecular binding of the C-terminal regions to the F2 and F3a sites. Our results suggest that the binding to the F3b site is blocked as a consequence of conformational changes. The potential interactions of the ligands with the ERMs in a cellular setting would thus be tightly regulated as most of the FERM domain ligands identified have class 3b motifs.

The present study contributes to the understanding of the ligand binding of one subgroup of FERM domains. The FERM family contains more than 50 additional members, and applying the identified principles on the whole family may allow us to learn more about conservation and variation of the binding pocket specificities.

## Supporting Information Available

Clonal phage ELISA for merlin FERM domain raw FP binding data related to Figure 3A; affinity determinations through competitive FP based assay for the FERM domains of the ERMs; energy landscape of the global docking simulation of the ZNF622_341–356_ peptide onto the moesin FERM domain; model of opening of the closed F3b of pocket of the moesin FERM domain generated by Rosetta Relax simulation; energy landscape of the global docking simulations on the F3 subdomain of known moesin FERM domain ligands (PDF)
Results of ProP-PD selections against ezrin FERM domain (XLSX)
Results of ProP-PD selections against radixin FERM domain (XLSX)
Results of ProP-PD selections against moesin FERM domain (XLSX)
Results of ProP-PD selections against merlin FERM domain (XLSX)
GO terms enriched for the combined ERM ligand set (XLSX)
Hot spot residues in the F3a and F3b binding sites of moesin FERM domain suggested by computational alanine scanning; affinities of peptides binding to moesin FERM M285A/H288A and moesin FERM K211A/I238A (PDF)
