# Supplementary material for: Elucidation of Short Linear Motif-Based Interactions of the FERM Domains of Ezrin, Radixin, Moesin, and Merlin
Source: Biochemistry. 2023 May 24;62(11):1594–607. doi: 10.1021/acs.biochem.3c00096 (PMC10249358; doi:10.1021/acs.biochem.3c00096)
Supplement: Supplementary file 7 — bi3c00096_si_007.pdf [file bi3c00096_si_007.pdf]

SUPPLEMENTAL TABLES S6-S7.

Table S6. Detection of binding hotspot residues in F3a and F3b binding sites using computational alanine scanning.

**A**	**F3b binders**					
	**MISP**		**TBX4**		**KIRREL**	
	DDG*	DG**	DDG	DG	DDG	DG
**M285A**	0.85	0.59	1.40	0.42	-	-
**H288A**	2.17	-0.06	3.14	-0.14	2.17	-0.01

**B**	**F3a binders**			
	**ZNF622**		**BTBD7**	
	DDG	DG	DDG	DG
**K211A**	1.59	-0.75	1.50	0.79
**I238A**	1.16	0.72	-	-
**F267A**	1.85	2.00	1.74	1.95

* DDG indicates the predicted change upon mutation to alanine in binding affinity

** DG indicates the predicted change upon mutation to alanine in stability.

Table S7: Affinities of peptides binding to moesin FERM M285A/H288A and moesin FERM K211A/I238A.

**Name**	**FITC-labeled probe**	**K_D (µM)**	
		**Moesin FERM M285A/H288A**	**Moesin FERM K211A/ I238A**
**LATS1$_{73\text{-}88}$**	THHKALQEIRNSLLPF	1.3 ± 0.1	7.4 ± 0.5
**EBP50$_{343\text{-}358}$**	APQMDWSKKNELFSNL-coo-	3.1 ± 0.2	114 ± 7
**ZNF622$_{341\text{-}356}$**	LEFADFYDFRSSYPDH	1.3 ± 0.1	46 ± 2
**KIRREL3$_{637\text{-}652}$**	YYSVNTFKEHHSTPTI	0.84 ± 0.05	0.17 ± 0.02
**TBX4$_{428\text{-}443}$**	SYSVQTMETVPYQPFP	6.9 ± 0.4	2.17 ± 0.31
**NOP53$_{192\text{-}207}$**	FYDLWASDNPLDRPLV	2.2 ± 0.1	42 ± 1
